# Patch-Clamp Single-Cell Proteomics in Acute Brain Slices: A Framework for Recording, Retrieval, and Interpretation

**DOI:** 10.1101/2025.09.15.675920

**Published:** 2025-09-17

**Authors:** Larry Rodriguez, Jolene Diedrich, Aline M. A. Martins, Le Sun, Blake Tsu, Stefanie Kairs, Roman Vlkolinsky, Christopher A. Barnes, Marisa Roberto, John R. Yates

**Affiliations:** 1 Department of Integrated Structural and Molecular Biology, The Scripps Research Institute, La Jolla, CA 92037; 2 NGeneBioAI, Inc. San Diego, CA 92121; 3 Yatiri Bio, Inc. San Diego, CA 92121; 4 Department of Translational Medicine, The Scripps Research Institute, La Jolla, CA 92037

**Keywords:** patch-clamp electrophysiology, single-cell proteomics, acute brain slices, ion channels, G protein-coupled receptors (GPCRs), synaptic proteins

## Abstract

Single-cell proteomics (SCP) is a powerful method for interrogating the molecular composition of neurons, yet its application to acute brain slices has remained limited. Patch-clamp electrophysiology provides direct information on neuronal excitability, synaptic inputs, and ion channel function, making it a natural partner for SCP. However, combining these techniques introduces unique challenges: neurons must be both physiologically characterized and physically collected, and variability during retrieval from the brain slice can affect how faithfully proteomic measurements reflect *in situ* physiology. Here, we introduce a framework for interpreting patch-SCP outcomes that considers retrieval quality in terms of both the amount of material collected and the synaptic contents being recovered. Using a shotgun strategy in which all patched neurons were collected regardless of electrophysiological outcome, we systematically benchmarked the retrieval of pyramidal neurons in the rat medial prefrontal cortex. Capacitance measured during gigaseal-preserved retrieval correlated with protein identifications, providing a proxy for linking soma size to proteome yield. Preservation of neuronal spiking during relocation was associated with broader synaptic enrichment and recovery of transmembrane proteins. By comparison, torn or aspirated neurons produced small proteomes with poor synaptic representation and neurons with lost gigaseals or no recordings displayed variable outcomes that could still yield substantial molecular information. Together, these results establish shotgun patch-SCP as both proof-of-concept and a practical framework for linking neuronal physiology with proteomics in semi-intact circuits.

## Graphical Abstract

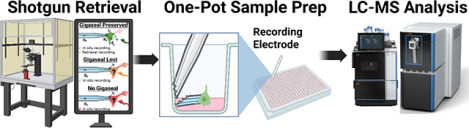

## Introduction

Whole-cell patch-clamp electrophysiology performed in acute brain slices is the gold standard approach for assessing the functional properties of individual neurons. In this configuration, a glass micropipette is used to establish electrical continuity with the intracellular space of a single neuron, which enables precise measurement of voltage- and ligand-gated ion channel activity, synaptic inputs, and intrinsic excitability. Advances in single-cell omics have extended the use of patch-clamp electrodes from measuring physiological responses to physically collecting neuronal material for molecular profiling. The first reported approach was RNA-sequencing, with “patch-seq” adoption following shortly thereafter.[1–4] More recently, progress in mass spectrometry-based single-cell proteomics (SCP) has opened the possibility of understanding patch-clamp recordings in the context of a neuron’s protein composition. Initial implementations of patch-SCP in intact brain slices relied on aspirating cytoplasmic contents through the recording electrode [5–7]. Subsequent work extended the approach to human iPSC-derived neurons, where whole somata could be collected and analyzed [8]. These advances illustrate rapid technical progress, but establishing a direct link between a neuron’s electrophysiological activity and its protein composition remains challenging on multiple fronts.

Augmenting the detailed neurophysiological insight gained from acute brain slice recordings requires SCP workflows that enable confident and consistent detection of the specific proteins within a neuron that influence its electrical properties. Electrophysiologists typically study ion channels, G protein-coupled receptors (GPCRs), and synapse-associated proteins, which mass spectrometrists recognize as challenging protein classes to detect because they are often low-abundance and membrane-embedded. This means that even when patch-clamp reveals distinct functional properties, the mass spectra may not capture the molecules that determine those signals. For example, a recent study of locus coeruleus noradrenergic neurons reported sex-specific differences in intrinsic excitability, but proteomic collection and analysis was largely limited to the cytoplasm, so the molecular basis for these differences remained unclear.[7] Similarly, a patch-SCP platform applied to Alzheimer’s disease hiPSC-derived neurons detected a hyperexcitable phenotype, yet the authors noted that many synaptic and membrane proteins were absent from the aspirated soma, leaving open the question of whether local synaptic activity (which is known to regulate protein transcription) was adequately captured.[8]

These studies highlight the feasibility of patch-SCP for linking neuronal physiology with molecular profiles, but they also illustrate important limitations: protein detection was biased toward cytoplasmic content, and key membrane or synaptic proteins that directly underlie electrophysiological properties were often absent. Beyond the challenge of detecting low-abundance, compartment-specific proteins, a fundamental limitation of patch-SCP involves the mechanical retrieval of neurons from brain slice preparations, which can be unpredictable. Indeed, proteomic recovery may fail if the soma is only partly collected (or tears) during withdrawal from the brain slice, or if material is lost during sample preparation. To minimize the impact of the retrieval problem, patch-SCP studies report an “all-or-nothing” approach, analyzing only neurons with stable recordings that yield a predefined minimum number of protein identifications within or across samples.[7, 8] While this strategy ensures high-quality electrophysiological data, it excludes a fraction of patched neurons and represents a bottleneck for assessing retrieval quality, optimizing sample handling, and expanding proteome coverage in brain slice preparations.

To overcome the limitations in patch-SCP, we developed a framework that explicitly evaluates how electrophysiological properties align with proteome recovery in single neurons from acute rat brain slices. We use this framework to assess the relationship between neuronal size and protein recovery, which was accomplished by maintaining the electrophysiological gigaseal as the neuron is retrieved from the slice. Although our goal was to obtain electrophysiological recordings during retrieval, we systematically collected neurons for proteomic analysis regardless of whether the high-resistance seal was formed in the initial neuron patching step or if it was formed but then lost before reassessment. Using this comprehensive “shotgun” strategy, we evaluated the retrieval process across all experimental patch-SCP outcomes, which allowed us to benchmark retrieval quality without imposing a bottleneck in sample collection. We profiled pyramidal neurons from Layer 2/3 (L2/3) of the rat medial prefrontal cortex (mPFC) because of the importance of this region in a variety of brain disorders, including anxiety, stress, and addiction [9, 10]. Our results demonstrate the feasibility of implementing our patch-SCP framework for detecting thousands of proteins per neuron (e.g. ion channels, GPCRs, and synaptic scaffolds) and integrating patch-clamp electrophysiology with SCP, which provides a pathway for method development and future studies on neuronal signaling in acute brain slices.

## Results and Discussion

### A Framework for Interpreting Patch-Clamp SCP Recordings

Whole-cell patch-clamp in acute brain slices is the definitive approach for investigating neuronal physiology because it provides direct access to the currents and potentials that govern excitability within a semi-intact circuit. After forming a high resistance “gigaseal” between the micropipette and soma, a patch of the neuron’s membrane is ruptured, which makes the electrode electrically continuous with the intracellular space (Supplementary Figure 1). This configuration allows measurement of intrinsic membrane properties such as capacitance (proportional to surface area or size of the neuronal soma) and resistance (R_M_, which reflects how effectively ionic current flow is restricted across the membrane and can be used as a proxy for neuronal integrity). The active membrane properties of the neuron can also be assessed, including the generation and pattern of action potentials (mediated by voltage-gated sodium, potassium, and calcium channels) as well as excitatory and inhibitory postsynaptic currents which reflect connectivity and ongoing network activity in the slice. Together, these define the electrophysiological “circuit” of the neuron whereby ion channel and synaptic activity give rise to neuronal excitability *in situ*. When the gigaseal is intact, this circuit can be interrogated in a stable and interpretable manner; without it, recordings are not possible due to electrical discontinuity with the soma. Thus, the rationale for combining SCP with patch-clamp is straightforward: to provide molecular context for the distinct electrophysiological properties of a neuron.

**Figure 1.**
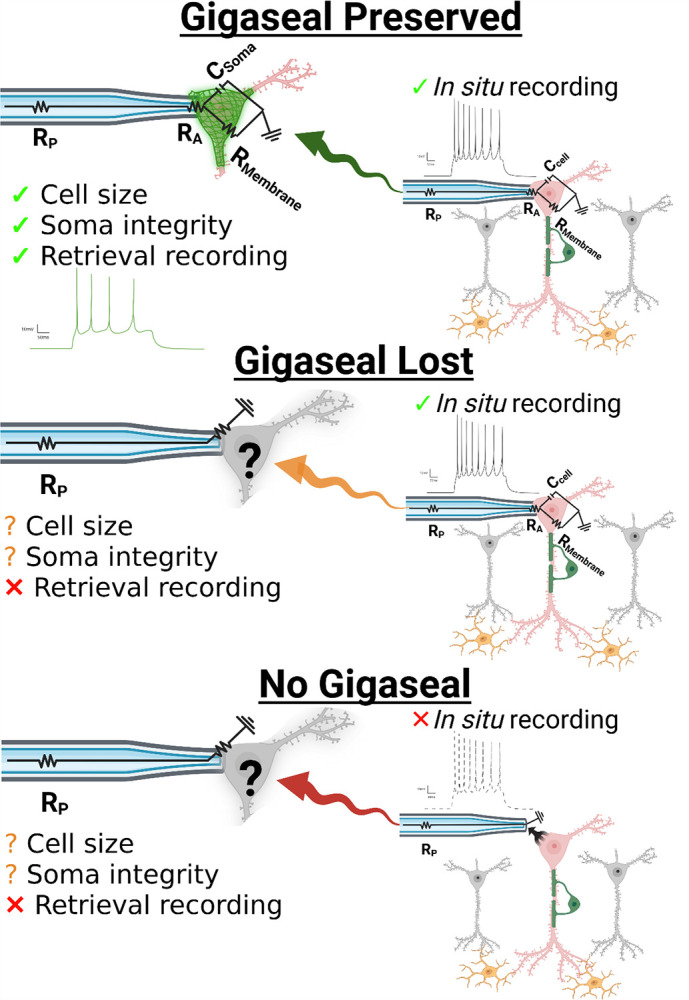
Conceptual framework for interpreting patch-SCP outcomes. Whole-cell patch-clamp provides access to a neuron’s intrinsic and synaptic properties, but retrieval introduces variability in how much of the soma (and its compartments) are recovered for proteomic analysis. Collection outcomes fall into three categories: (i) gigaseal preserved during retrieval, enabling continued electrophysiological monitoring of soma properties, (ii) gigaseal lost during retrieval, leaving pre-retrieval recordings valid but with uncertain proteome recovery, or (iii) no gigaseal, in which somata can still be collected but without functional characterization. Abbreviations: R_p_, pipette resistance; R_A_, access resistance; R_M_, membrane resistance; C_cell_, cell capacitance. Created with BioRender.

A critical consideration for performing patch-SCP in acute brain slices is the process by which a neuron is retrieved or extracted. Incomplete collection may cause protein loss that detaches the mass spectrometry readings from electrophysiological responses *in situ*. For example, a neuron may display robust sodium channel-dependent spiking, yet if the soma is only partially collected, the Na_V_ subunits that mediate this response may not be detected by the mass spectrometer. Such “false negatives” can complicate interpretation, particularly for low-abundance transmembrane proteins like ion channels, which are also difficult to extract, digest, and detect by traditional proteomic workflows.[11] Considering these challenges, we hypothesized that maintaining a stable gigaseal during retrieval would help reduce the uncertainty associated with the collection process. Figure 1 illustrates that, by preserving the electrical accessibility of the soma as it is withdrawn from the brain slice, properties like capacitance, resistance, and excitability can be reassessed during collection, providing surrogate measures for how much neuronal material is collected from the brain slice and the quality of the retrieval process. Likewise, if the gigaseal is lost during retrieval, then the acute slice recordings performed *in* situ may still be used to differentiate between brain region-specific neuron-types, but then proteome recovery lacks context. In the case of poor-quality retrievals, sample loss could undermine the ability to link electrophysiological properties with their molecular elements. A final category of neurons is those that fail to form a gigaseal, which provide no electrophysiological data and are often excluded from proteomic analysis.[7, 8] Given the uncertain nature of neuron retrieval, we reasoned that neurons lacking electrophysiological recordings could still be useful for benchmarking proteome recovery, assessing mass spectrometry performance, and validating sample processing.

Our framework formalizes the challenges of recovering low-abundance or compartment-specific proteins (highlighted in earlier patch-SCP studies[6–8]) as interpretive boundaries: electrophysiology provides “true-positive” measurements of ion channel and synaptic function, but the stochastic nature of retrieval can detach protein identification(s) from those signals. Recognizing this limitation is essential. If an ion channel subunit is not detected despite clear electrophysiological evidence of its activity, the most rigorous explanation is technical loss during retrieval rather than biological absence. Accordingly, patch-SCP interpretations should be proportional to both the sensitivity of the method and the biological question under investigation.

### Gigaseal Preservation: A Quantitative Bridge Between Proteomics and Electrophysiology

**Figure 2.**
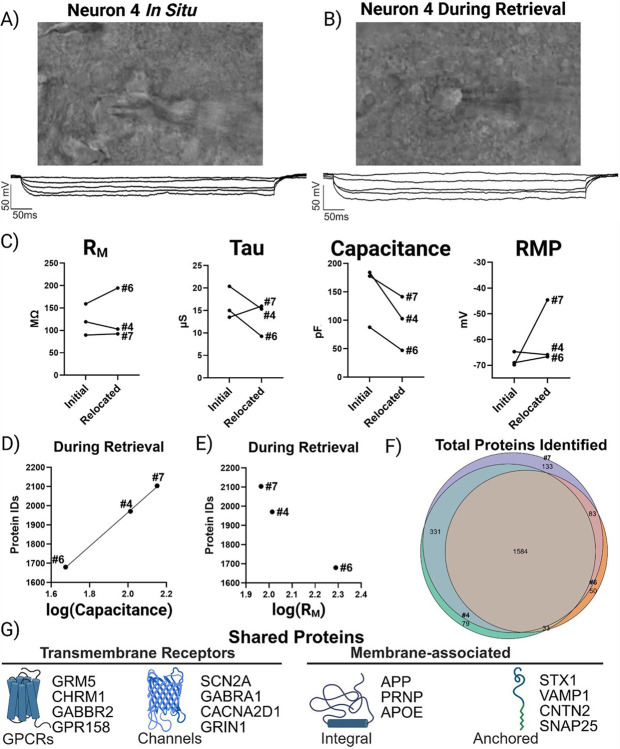
Gigaseal preservation links neuron size to proteome recovery. **(A) DIC image and** current clamp recording of neuron #4 before retrieval (i.e. *in situ*). (B) Neuron #4 relocated to the slice surface while maintaining whole-cell access. (C) Ladder plots depicting a change in selected passive membrane properties from initial recordings and during retrieval of each neuron (n = 3). Passive properties were determined for each individual neuron from linear fits and extrapolated at I = 0 pA using NeuroExpress.[12] (D) Protein identifications correlated with log-transformed capacitance, linking soma size to proteome yield. (E) No correlation was observed with log-transformed membrane resistance (R_M_). (F) Venn diagram showing a core proteome (>1500 proteins) shared across gigaseal-preserved neurons. (G) Examples of consistently recovered proteins, including *APP*, *PRNP*, *APOE*, *SCN2A*, *GRIN1*, and *GRM3*.

To test our patch-SCP framework, we sought to maintain the gigaseal during neuron relocation and determine if recordings measured under this condition could predict proteome recovery (i.e. protein identifications). Figure 2A shows a representative image of neuron #4 and its current clamp recording in a brain slice, taken prior to the initiation of the retrieval process. These *in situ* current clamp recordings can provide valuable neurophysiological information (e.g. cell-typing by AP firing patterns) but have not been reported to provide insight into the quantity or quality of neuron retrieval (e.g., soma size and neuron integrity or viability once withdrawn). Figure 2B shows that careful relocation of the neuron to the slice surface did not disrupt the whole-cell configuration (n = 3), as evidenced by stable hyperpolarizing steps. The passive membrane properties for each neuron were initially assessed *in situ* (i.e. in the acute brain slice) and then reassessed during retrieval near the slice surface (Figure 2C). Our results show that, while physiological changes can occur during retrieval, the gigaseal can be maintained well enough to reassess passive properties, such as resting potential (RMP; a proxy for health), capacitance (proportional to soma size), and resistance (R_M_; associated with membrane integrity). Importantly, total protein identifications correlated with the log-transformed capacitance (F = 1577, p < 0.05, adjusted R^2^ = 0.998, n = 3), providing a direct link between soma size and proteome recovery (Figure 2D). In contrast, log-transformed R_M_ showed no significant correlation with protein identifications (F = 0.748, p > 0.05; Figure 2E). These results indicate that soma size, which is proportional to capacitance, plays a more direct role in protein recovery than R_M_, which primarily reflects the ability of ions to move across the surface of the neuronal membrane (and is inversely proportional to size). Over 1500 proteins were identified between these neurons, and at least 1700 total proteins were detected in each (Figure 2F). This consistent ‘core’ proteome included membrane-associated proteins (*APP*, *PRNP*, *APOE*), ion channel subunits (*SCN2A*, *CACNA2D1*, *GABRA1*, *GRIN1*), and GPCRs (*GRM3*, *GRM5*, *CHRM1*) (Figure 2G; Supplementary Data 1) indicating that our SCP workflow is capable of sensitive and robust protein detection.

**Figure 3.**
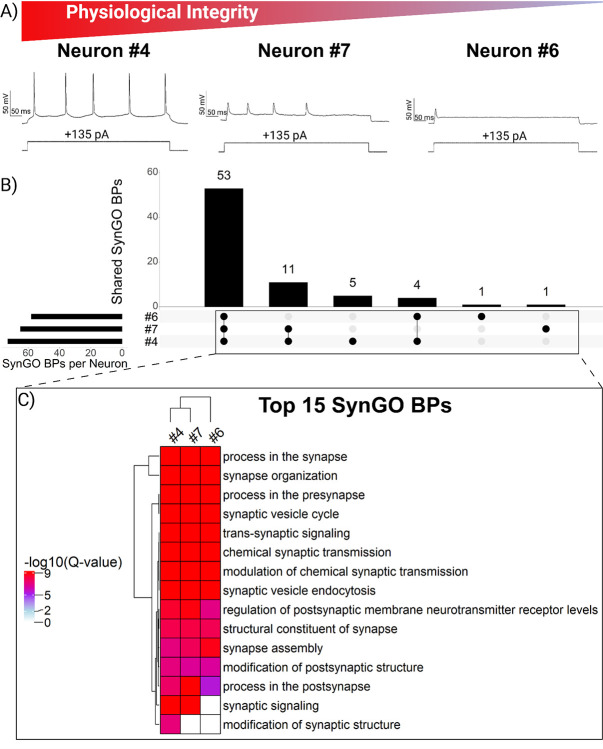
Preservation of active properties during retrieval is associated with recovery of synaptic proteins. (A) Action potential firing profiles illustrating differences in retrieval quality: neuron #4 retained stable spiking, neuron #7 showed reduced amplitude consistent with leak, and neuron #6 produced only a single spike following partial aspiration. (B) UpSet plot quantifying overlap of synaptic biological process (BP) terms (annotated by SynGO) across each neuron. (C) Heatmap of top BP terms, sorted by enrichment significance. Color intensity reflects statistical significance (−log_10_ adjusted Q-value; Q-value < 0.05 cutoff). Neuron #4 exhibited the greatest diversity of enriched terms, while neuron #6 lacked significant enrichment for synaptic signaling, despite many protein identifications.

We next asked whether preserving the integrity of active membrane properties during retrieval would correlate with enhanced recovery of proteins with distinct neuronal functions and biological insight (i.e. recovery of proteins with diverse functions, rather than the sheer number of distinct protein identifications). We hypothesized that neurons retaining the ability to fire action potentials despite mechanical relocation would be enriched for proteins underlying synaptic signaling and neuronal identity. As proof of principle, we found that relocating neurons to the surface of the slice did not prevent depolarizing currents from inducing action potentials (Figure 3A), demonstrating that active membrane properties could often be preserved during retrieval. At the same time, the stochastic nature of retrieving neurons from an acute brain slice is reflected by variation in the integrity of neuronal spiking. Neuron #4 represents an ideal retrieval: a large, stable cell in which depolarizing steps reliably induced consistent action potentials. By comparison, retrievals of neurons #6 and #7 were more challenging and both displayed altered active properties likely reflecting membrane disruption or damage near the axon hillock. Neuron #7 gently entered the electrode, but still generated several action potentials during depolarizing steps, albeit with reduced amplitude, which is consistent with leak currents and a compromised membrane. In contrast, the partial aspiration of neuron #6 during whole-cell configuration produced a more drastic outcome, with current-clamp recordings showing only a single spike during depolarization.

To evaluate the impact of neuron retrieval quality (as reflected by spike integrity) on the recovery of proteins with distinct neuronal functions, we utilized SynGO, a curated database tailored for analyzing gene ontologies of compartments and biological processes specific to synapses.[13] SynGO analysis revealed 53 biological processes (BPs; Figure 3B) and 24 cellular components (CCs; Supplementary Data 2) significantly enriched (Q-value < 0.05) across the proteomes of neurons retrieved under a gigaseal. Consistent with our finding that neuron size influences protein recovery, the smallest neuron (#6) exhibited the fewest enriched cellular component terms (27, versus 32 in #4 and 29 in #7). A similar trend was observed for biological processes (BPs), where the well-retrieved large neuron (#4) showed the greatest diversity of GO terms. Neurons #4 and #7 shared many features, including overlapping protein identifications (Figure 2F), detection of somatostatin (Supplementary Data 1), enrichment of BPs such as synaptic signaling and organization (Figure 3B), and recovery of proteins from specialized CCs such as the active zone and anchored synaptic membrane components (Supplementary Data 2). However, despite being the largest neuron by both electrophysiological and proteomic measurements, GO analysis of neuron #7 produced the fewest unique BP terms. Interestingly, the neuron with the most compromised active properties (#6) lacked significant enrichment for proteins associated with synaptic signaling (Q-value > 0.05), despite detection of several ion channel subunits such as SCN2A and GABRA1 which are involved in neurotransmission. Together, these results suggest that electrophysiological properties which reflect neuron size (i.e. capacitance) can guide interpretation of proteomic recovery and challenge the common assumption that higher protein counts necessarily reflect deeper biological insight. Instead, our data indicates that the quality of neuron retrieval (reflected in the integrity of neuronal spiking during relocation), is closely associated with the recovery of synaptic proteins.

### Retrieval Loss Decouples Proteomic Measurements from Electrophysiology Recordings

**Figure 4.**
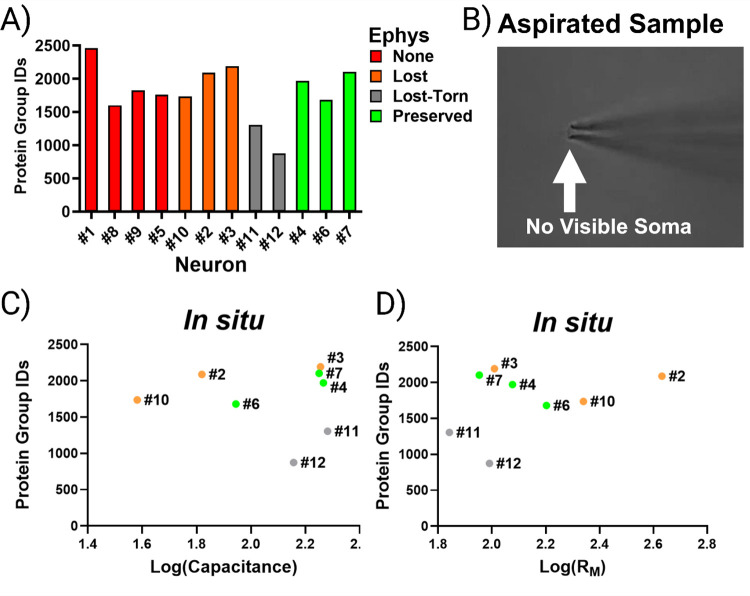
Proteome recovery is not associated with recordings performed *in situ*. **(A)** (A) Protein identifications across all neurons, grouped by patch outcome. (B) Example of a “torn” neuron that was aspirated during retrieval, yielding few proteins. (C-D) Log transformations of capacitance and R_M_ measured prior to retrieval did not correlate with protein identifications (p > 0.05, n = 8). Colors denote the success-level of electrophysiological characterization during the course of the patch-SCP process.

Our findings suggest that preserving electrophysiological access to the neuron during patch-SCP retrieval provides a quantitative anchor for interpreting proteomic recovery. However, because this configuration is challenging to achieve in acute brain slices, not every neuron can be successfully collected this way. Therefore, we asked whether proteomic recovery could be predicted by electrophysiological measurements made prior to neuron retrieval from the slice. Figure 4A summarizes the protein identifications for all neurons, which can be classified by the success or failure of establishing the whole-cell patch-clamp configuration and further grouped by when the gigaseal was last intact, before or during neuron retrieval. Neurons that were torn or aspirated into the electrode during retrieval (Figure 4B) produced the fewest detected proteins of all categories, despite having been characterized by electrophysiological recordings in the brain slice. The analysis of known poor-quality retrievals like these can serve as important negative controls and demonstrate that even robust recordings cannot compensate for severe retrieval loss. Analysis of passive membrane properties measured prior to retrieval from the brain slice showed that neither log-transformed capacitance nor R_M_ correlated with protein identifications (p > 0.05; n = 8; Figures 4C–D). These results indicate that the amount of somal material being recovered from the brain slice does not reflect the electrophysiological recordings performed before neuron collection.

While recordings performed *in situ* provide valuable physiological insight, mechanical retrieval can introduce sample loss that uncouples the proteome from these properties. By comparison, retrievals without a gigaseal (i.e. somata isolated by micropipette without recordings) also suffered from variability but could yield similarly large proteomes, with 1,400–2,300 proteins detected per cell. These results suggest that our SCP-MS workflow is sensitive enough to recover thousands of proteins from any neuron that remains visibly intact at the pipette tip and highlights the value of including all retrieval outcomes in analysis.

### Comprehensive Neuron Retrieval: A Strategy for Benchmarking SCP Performance

Recognizing that retrieval loss has the potential to uncouple SCP from patch-clamp recordings, we then asked whether proteomic analysis of all samples could help identify characteristics of high- versus low-confidence retrievals. Rather than discarding cells that do not meet conventional patch-clamp criteria, we were interested in comparing proteome recovery across retrieval categories and determining whether structures in the data emerged.

**Figure 5.**
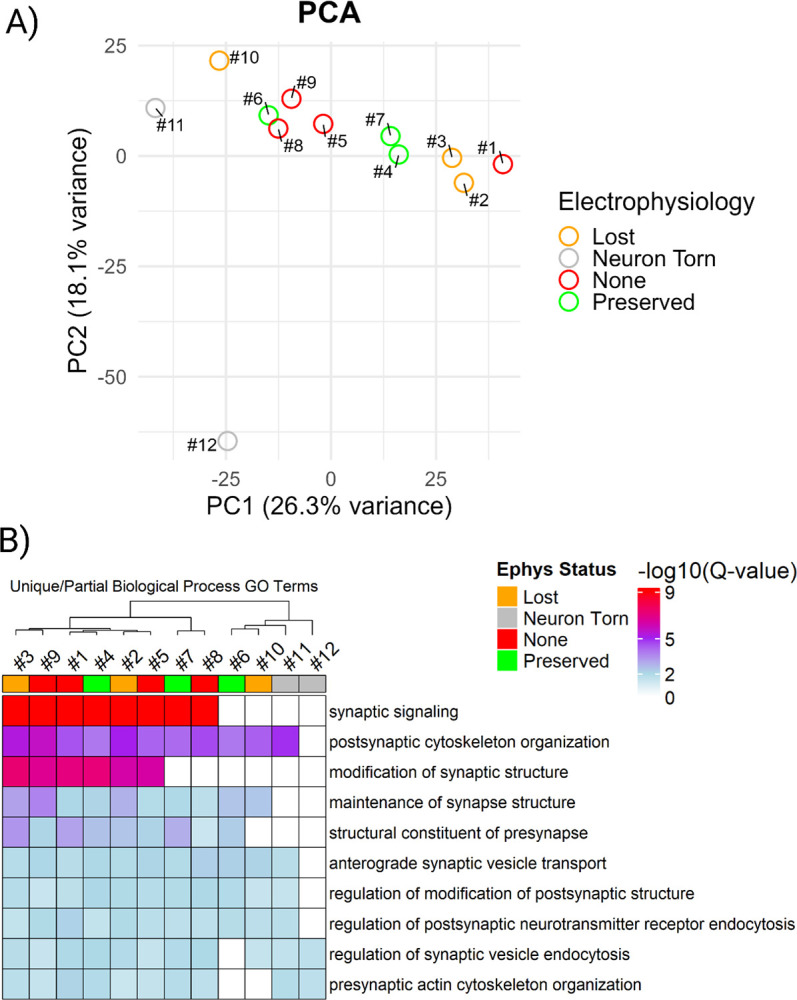
Comprehensive analysis can distinguish between high- and low-confidence retrievals. (A) PCA of single-neuron proteomes revealed clustering by retrieval quality rather than by strict patch outcome. Torn neurons (grey) separated from others, while neuron #6 (gigaseal but poor spiking) and neuron #10 (gigaseal lost during retrieval) clustered with cells failed to form gigaseals (red). (B) Heatmap of SynGO-enriched biological processes highlighting that synaptic enrichment varied across neurons, independent of outcome category.

Principal component analysis (PCA) of all neurons (Figure 5A) revealed clear separation of extreme cases: torn neurons (#11 and #12, grey) clustered apart from all others, consistent with their poor retrieval, which was confirmed visually. Neuron #6, retrieved under a gigaseal but with compromised active properties, grouped more closely with neurons lacking gigaseals (#5, #8, #9; red), suggesting that its proteome resembled a coarse retrieval: large in terms of protein identifications but lacking key qualitative features, such as enrichment for synaptic signaling. Conversely, neuron #1 (no gigaseal) more closely resembled neurons with successful initial brain slice recordings (#2 and #3; orange). These results suggest that proteomic profiles contain qualitative structures that do not strictly adhere to categorical patch-clamp outcomes.

To determine whether the proteomic structures observed across retrieval outcomes reflected differences in biological insight, we performed GO analysis on the proteome of each neuron using SynGO.[13] Across all neurons, we identified a total of 23 cellular components and 45 biological processes that were consistently enriched (Supplementary Figure 2; Q-value < 0.05). Detection of a core set of synaptic processes indicates that consistent proteomic coverage by patch-SCP is possible when three factors converge: (i) meticulous neuron retrieval, (ii) sample preparation methods that minimize loss, and (iii) the sensitivity of high-performance DIA mass spectrometry. Notably, SynGO enrichments did not segregate strictly by patch-clamp outcome or by the number of identified proteins, which suggest that a proteome-centric approach could be useful for assessing the quality of patch-SCP interpretation. Analysis of partially shared biological processes revealed that several neurons (#6 and #10) lacked significant enrichment for synaptic signaling despite being characterized by electrophysiology (Figure 5B). By clustering with torn neurons (#11 and #12), which serve as internal standards for poor retrieval, these results highlight that stochastic variability during retrieval limits the recovery of synaptic information. This underscores the importance of comprehensive inclusion: a large number of protein identifications alone cannot guarantee biological insight, whereas analyzing all retrieval outcomes provides the context needed to distinguish high- from low-confidence proteomes. Together, these results suggest that retrieval quality is best understood as a spectrum with two dimensions: a quantitative axis reflecting the amount of material collected (e.g., soma size and protein yield) and a qualitative axis reflecting which compartments are preserved (e.g., synaptic enrichment, ion channel recovery) (Supplementary Figure 2A).

Developing new patch-SCP methods can require fast turnaround and iterative optimization of retrieval technique, sample preparation, and MS acquisition. In this context, proteome-centric analysis of all neurons provides a practical alternative, because even when electrophysiological characterization is missing, these datasets can inform method development by benchmarking recovery and process-related losses. Our findings indicate that the stochastic nature of the retrieval process can be partially revealed by a comprehensive analysis of all patch outcomes, where comparisons across categories provide the context to identify high- versus low-confidence retrievals. We emphasize that neurons characterized by proteomics alone are not a substitute for those with electrophysiological data; rather, we suggest that the inclusion of all samples serves two purposes in patch-SCP: (i) to benchmark retrieval (given somata are visible) and sample-handling performance against proteomic readouts, and (ii) to supplement molecular information that can corroborate hypotheses (e.g., pathway enrichment or receptor detection) when functional data are limited.

### Profiling Transmembrane Proteins With SCP

Under the proposed framework, the detection of ion channels and GPCRs represent the most important targets for patch-SCP. Therefore, we wanted to assess whether our patch-SCP workflow was robust enough to detect low abundance and hydrophobic transmembrane proteins.

**Figure 6.**
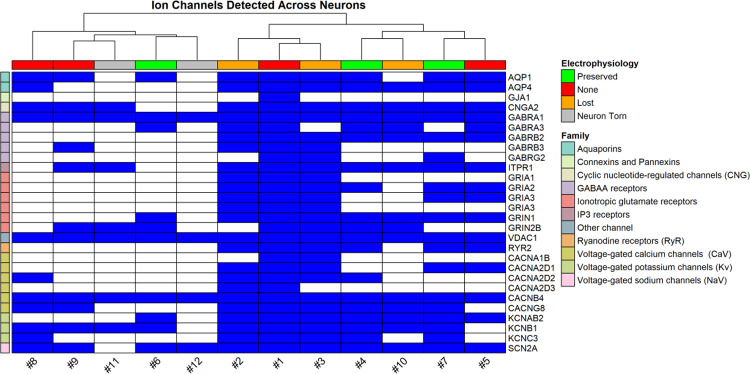
Recovery of ion channels across neurons retrieved by patch-SCP. Binary heatmap showing detected ion channel subunits (blue) across individual neurons. Rows are grouped by channel family (e.g., sodium, calcium, glutamate, GABA). Columns are annotated by the success-level of electrophysiological characterization during the course of the patch-SCP process. Neurons with gigaseals generally retained broader sets of ion channel subunits, although some gigaseal-lost neurons recovered more subunits than preserved ones. Torn neurons consistently showed the poorest recovery.

We assessed the detection of membrane receptor proteins in each sample by creating ion channel and GPCR recovery lists, using a combination of SynGO annotations and curated gene tables from IUPHAR-DB, a peer-reviewed resource of pharmacologically relevant protein families [14, 15]. Figure 6 illustrates that our workflow detected diverse classes of ligand- and voltage-gated ion channels, including GABA_A_ receptor subunits (e.g., *GABRA1*, *GABRB2*, *GABRG2*), AMPA and NMDA receptor subunits (*GRIA1–3*, *GRIN1*, *GRIN2B*), and multiple components of Ca_v_2.2 channels (*CACNA1B*, *CACNA2D1–3*, *CACNB4*, *CACNG8*). Promisingly, we were able to identify several ion channel subunits with distinct functional roles across samples, regardless of patch-clamp retrieval category. In all neurons, we detected the α subunit of the ligand-gated GABA_A_ ion channel, which produces inhibitory postsynaptic currents (IPSCs), as well as *CACNB4* the auxiliary β subunit of the voltage-gated Ca_v_2.2 channels, which regulate excitability and neurotransmitter release.[16] The voltage-dependent anion channel *VDAC1* was also detected in all neurons, although this protein is associated with the maintenance of a potential gradient across the outer mitochondrial membrane and is not expected to be in as low-abundance as ion channels found on the plasma membrane.[17] *SCN2A*, the alpha subunit of Na_v_1.2 that initiates AP propagation [18], was detected in all neurons except #11, which was torn during retrieval. Its near-universal detection is consistent with its expected expression within the axonal initial segment and across CNS neurons.[19] We also observed peptide mapping to transient receptor potential channels, though ambiguous assignments precluded its confident inclusion in Figure 6.

Overall, ion channel and GPCR (Supplementary Figure 3) detection patterns seemed to vary by retrieval quality. Higher-quality retrievals often but not exclusively included neurons with initial electrophysiological recordings and tended to retain a broader diversity of transmembrane proteins, while torn or severely compromised neurons consistently showed reduced recovery. Ion channel and GPCR recovery did not always scale with electrophysiology success at the time of retrieval; in some cases, more transmembrane proteins could be found among neurons that lost the whole-cell configuration during retrieval than neurons with continuous whole-cell access. This could reflect variability in the recovery of membrane compartments, as ion channels are both low in abundance and spatially restricted, although our sample size is insufficient to determine whether these differences are technical or biological. Regardless, even neurons lacking electrophysiological recordings showed consistent detection of several ion channel subunits, underscoring the value of including all retrieved cells. While such samples cannot replace functional recordings, when coupled with high-sensitivity DIA MS/MS workflows, their proteomes can provide supplemental molecular context.

### Limitations and Future Directions

Our data indicates that patch-SCP retrieval is best understood along two dimensions. A quantitative axis reflects the amount of neuronal material collected, as indicated by the correlation between capacitance measurements during neuron retrieval and protein identifications. At the same time, a qualitative axis reflects which compartments are preserved during retrieval, as indicated by the enrichment of synapse-specific processes and the recovery of transmembrane proteins. Each axes seems to reflect a distinct set of electrophysiological properties. For example, the proteome recovered from neuron #6 was proportional to its size but lacked enrichment for synaptic signaling, consistent with partial loss of specialized compartments during retrieval. Building on previous patch-SCP studies [6–8] which primarily categorized recovery outcomes as binary, our results suggest that outcomes are better interpreted as a spectrum in which both axes shape (or limit) the biological insight that can be gained. We note that interpretation of GO enrichments at the single-cell level requires caution. While our data reproducibly captured synaptic compartments and processes, some neurons with otherwise large proteomes lacked enrichment for synaptic signaling. This can reflect both biological variability and stochastic retrieval, which is why GO analysis in patch-SCP is best considered ambigous until larger datasets are available to provide deeper context.

A major limitation in patch-SCP arises from the mechanics of soma withdrawal. Physical disruption of the axon hillock or proximal dendrites may prevent complete recovery of synaptic and membrane proteins, even when the whole-cell configuration is maintained. This likely contributes to the incomplete or variable enrichment of synaptic processes in some neurons and as highlighted by others[6, 7], improved retrieval methods could help preserve neuronal architecture. It is important to note that the unusually large proteome observed in neuron #1 seemed consistent with its observed soma size and could be due to its proximity to the slice surface. While this underscores the impact of retrieval geometry on proteome recovery, further work is required to determine whether such neurons should be considered representative or exceptional cases. Culture-based patch-SCP studies [8] may mitigate this issue because cell retrieval is technically straightforward, although they lack the intact circuit context that acute slice preparations provide.

Although we detected subunits of major ion channel complexes (e.g., *SCN2A*, *CACNA2D1–3*, *GABRA1*), complete functional assemblies were not consistently recovered. Similarly, several GPCR families of high biological interest (opioid, adrenergic, serotonergic, and CRF_1_) were not detected, although promisingly, neuropeptides such as somatostatin (SST) and VGF could be recovered. This reflects both the low abundance of certain subunits and the technical challenges of detecting hydrophobic proteins. Prior reports noted limited coverage of plasma membrane receptors [6–8] and our own results underscore that recovery of full complexes remains a key barrier for patch-SCP. Similarly, our electrophysiology protocol was limited to current clamp, which provides information about intrinsic excitability but not direct ion channel conductance. Expanding to voltage clamp and pharmacological approaches will be required to align functional recordings of specific channel types with their proteomic detection (e.g. inferring channel stoichiometry directly from proteomic data). Recovery of more challenging targets will likely require further optimization of retrieval techniques, sample preparation, and DIA acquisition strategies. Future iterations of patch-SCP could incorporate chemical probes or affinity tags into the patch pipette, building on precedents where small molecules (e.g., biocytin) are delivered during recording to label neuronal morphology.[5, 7] Such approaches may enable selective enrichment of membrane or synaptic proteins, which remain challenging targets in single-cell proteomics. Multiplexed proteomic strategies (e.g., TMT-based [6]) could also be adapted to patch-SCP, provided that single-cell specificity is preserved.

Despite these limitations, our study establishes a framework for balancing deep functional characterization with comprehensive molecular profiling. By treating retrieval as a spectrum and systematically including all outcomes, patch-SCP can be tailored to support applications in neuronal subtype classification, pharmacological perturbations, and disease-relevant investigations in acute brain slice preparations.

## Conclusions

Shotgun patch-SCP provides a framework for integrating electrophysiological and proteomic measurements at the single-neuron level in acute brain slices. By monitoring neuronal properties during retrieval, we show that parameters such as capacitance and spike integrity serve as indicators of proteome recovery. Our results demonstrate that retrieval mechanics, rather than *in situ* physiology alone, dictate whether proteins underlying excitability and synaptic function are recovered. Recognizing this constraint motivated a comprehensive inclusion strategy, where analyzing all patch outcomes provides context, benchmarks retrieval quality, and prevents overinterpretation of protein counts. At the same time, incomplete recovery of certain membrane proteins highlights a need for further optimization of retrieval and sample preparation. Together, these findings establish shotgun patch-SCP as both proof-of-concept and a flexible platform that can support future applications, from neuronal subtype classification to pharmacological studies and disease-relevant investigations in semi-intact brain circuits.

## Methods and Materials

### Electrophysiology

All animal studies conformed to NIH Guidelines and were approved by the TSRI IACUC. Wistar rats (75 days of age, Charles River) were given access to food and water ad libitum. Acute brain slices and electrophysiological recordings were performed as previously described [9, 20–24]. Briefly, rats were anesthetized with isoflurane before cervical dislocation and surgical brain isolation. 300 um coronal mPFC slices (300 μm) were prepared in an ice-cold high-sucrose cutting solution (sucrose 206 mM; KCl 2.5 mM; CaCl_2_ 0.5 mM; MgCl_2_ 7 mM; NaH_2_PO_4_ 1.2 mM; NaHCO_3_ 26 mM; glucose 5 mM and HEPES 5 mM) using a Leica VT1200S vibratome. Slices were incubated in 95% O_2_/5% CO_2_ equilibrated artificial cerebrospinal fluid (aCSF; NaCl 130 mM; KCl 3.5 mM; NaH_2_PO_4_ 1.25 mM; MgSO_4_·7H_2_O 1.5 mM; CaCl_2_ 2.0 mM; NaHCO_3_ 24 mM and glucose 10 mM), first at 37°C for 30 minutes, then at room temperature for another 30 minutes. Slices were then transferred to a recording chamber (Warner Instruments) and superfused at a flow rate of 2–4 ml/min. Whole-cell patch-clamp recordings were performed in neurons present in the medial subdivision of the PrL and clamped at −70 mV using a Multiclamp 700B amplifier, Digidata 1440A and pClamp10. Patch pipettes (3–6 MΩ) were filled with an internal solution composed of the following (in mM): 145 KGluconate, 0.5 EGTA, 2 MgCl_2_, 10 HEPES, 2 Mg-ATP and 0.2 Na-GTP. The intrinsic membrane properties and excitability of L2/3 neurons were determined in aCSF in current-clamp mode using a step protocol comprised of 500 ms hyperpolarizing and depolarizing steps in 5 or 10 pA increments (see [25]) and analyzed using the NeuroExpress software (version 19.4.09.) developed and provided by A. Szücs.[12] During withdrawal of the patched neuron, light negative pressure (−50 to −140 mmHg) was applied through the pipette and adjusted to facilitate soma retrieval while preserving the high-resistance seal. This gentle suction helped maintain gigaseal integrity as the soma was relocated toward the slice surface. Following retrieval, an image of the pipette tip was taken above the slice, to confirm whether an intact soma was present before proceeding to sample processing.

### Sample Processing and LC-MS/MS Acquisition

Micropipettes containing the retrieved neurons were immediately transferred to a 384-well non-binding microplate (Greiner) containing 15 μL of 0.02% n-dodecyl-β-D-maltoside (DDM) in UHPLC-grade water (ThermoFisher). To release the soma and its contents into the surfactant solution for subsequent processing, the pipette tip was carefully snapped in the well plate, which was stored on dry ice throughout the collection process. Samples were digested at 37°C for 2 hours using 7 ng of sequencing-grade Trypsin (Promega), quenched with 2 μL of 0.1% formic acid, and stored at −20°C for later processing. Peptide separation was performed using a Vanquish Neo UHPLC system (ThermoFisher) coupled to a 25 cm × 75 μm IonOpticks Aurora XS column with an integrated emitter. Samples were loaded directly and separated using a 36-minute gradient at 400 nL/min. A large wash cycle was included between runs to minimize carryover. MS analysis was performed on an Orbitrap Astral mass spectrometer (ThermoFisher Scientific) operating in data-independent acquisition (DIA) mode with FAIMS Pro using a single compensation voltage (CV = −50). Survey scans were acquired at 240,000 resolution (at m/z 200) over a mass range of 400–1000 m/z. DIA windows were 20 m/z wide with overlapping edges. MS1 AGC target was set to 800%, with a maximum injection time of 50 ms. Fragmentation was performed using higher-energy collisional dissociation (HCD) with normalized collision energy (NCE) of 25.

### Bioinformatic and Statistical Analysis

Raw DIA files were analyzed using DIA-NN v1.8.1 in library-free mode with the “match-between-runs” option enabled.[26] Searches were performed against the UniProt Mus musculus reference proteome (downloaded 2024), with oxidation selected as a variable modification. Up to two missed tryptic cleavages were allowed. Decoys were generated using the default reversed-sequence approach. Protein identifications were filtered at 1% false discovery rate (FDR) at both the precursor and protein group level. For all downstream analysis, only protein groups that passed both filters (i.e., those appearing in DIA-NN’s report.pg_matrix.tsv) were retained. Proteomic quantification was performed using DIA-NN’s MaxLFQ implementation, which integrates peptide-level signals into protein-level intensity estimates across samples. Enrichment terms for cellular component (CC) and biological process (BP) categories were derived from SynGO [13], a manually curated synaptic GO ontology database. Gene set enrichment analyses were performed on gene lists derived from DIA-NN’s protein-level output. For SynGO analysis, proteins were annotated based on gene symbol and GSEA filtering was performed under stringent conditions. The threshold for statistical significance for SynGO analyses was Q-value of < 0.05. Per-cell gene lists were extracted from report.pg_matrix.tsv using detected/not detected thresholds after 1% protein-level FDR filtering. GO term enrichment and clustering analyses were conducted using R 4.3.1 and visualized with packages including ComplexHeatmap, ggplot2, and UpSetR. Custom scripts for figure generation and SynGO-based analysis are available at https://github.com/LarryThePharmacologist. Ion channels and GPCRs annotation lists were generated using curated gene families from SynGO [13] and IUPHAR-DB [14, 15]. The raw mass spectrometry data and search files have been deposited to the ProteomeXchange Consortium (http://proteomecentral.proteomexchange.org) via the MassIVE partner repository with the MassIVE dataset identifier MSV000099156 and ProteomeXchange dataset identifier PXD068359.

## Supplementary Material

Supplement 1

Supplement 2

1

## Abbreviations

SCP: single-cell proteomics
patch-SCP: patch-clamp single-cell proteomics
DIA: data-independent acquisition
MS: mass spectrometry
MS/MS: tandem mass spectrometry
hiPSC: human induced pluripotent stem cell
mPFC: medial prefrontal cortex
GPCR: G protein-coupled receptor
NaV: voltage-gated sodium channel
CaV: voltage-gated calcium channel
AMPA: α-amino-3-hydroxy-5-methyl-4-isoxazolepropionic acid receptor
NMDA: N-methyl-D-aspartate receptor
GABA: γ-aminobutyric acid
IPSC: inhibitory postsynaptic current
AP: action potential
C_Cell_: capacitance
Rm: membrane resistance
Rp: pipette resistance
Ra: access resistance
CC: cellular component
BP: biological process
GO: Gene Ontology
VDAC: voltage-dependent anion channel
SST: somatostatin
CRF1: corticotropin-releasing factor receptor 1
