# Supplementary material for: Patch-Clamp Single-Cell Proteomics in Acute Brain Slices: A Framework for Recording, Retrieval, and Interpretation
Source: bioRxiv. 2025 Oct 14:2025.09.15.675920. Originally published 2025 Sep 17. Preprint. [Version 2] doi: 10.1101/2025.09.15.675920 (PMC12458218; doi:10.1101/2025.09.15.675920)
Supplement: Supplement 1 [file media-1.pdf]

Patch-Clamp Single-Cell Proteomics in Acute Brain Slices: A Framework for Recording, Retrieval, and Interpretation

Larry Rodriguez[1], Jolene Diedrich[1], Le Sun[1], Blake Tsu[2,3], Stefanie Kairs[2,3], Roman Vlkolinsky[4], Christopher A. Barnes[2,3], Aline M. A. Martins[1], Marisa Roberto[4], and John R. Yates 3rd[1]*

[1] Department of Integrated Structural and Molecular Biology, The Scripps Research Institute, La Jolla, CA 92037

[2] NGeneBioAI, Inc. San Diego, CA 92121

[3] Yatiri Bio, Inc. San Diego, CA 92121

[4] Department of Translational Medicine, The Scripps Research Institute, La Jolla, CA 92037

* Corresponding Author

Legends

Table S1 (XLSX). Protein-level DIA-NN1.8.1 output (report.pg_matrix.tsv). Contains gene names, quantification values, and associated data for all analyzed neurons.

Table S2 (XLSX). SynGO Biological Process (BP) enrichment results for each neuron. Each column corresponds to a neuron, with rows representing enriched GO terms and statistical values. Stringent evidence filter selected.

Table S3 (XLSX). SynGO Cellular Component (CC) enrichment results for each neuron, structured as in Table S2.

Supplementary Figure 1. Schematic of the patch-clamp process. Diagram illustrating the transition from initial pipette contact ("Patch attempt", top left) to full whole-cell configuration (top right), which allows simultaneous electrical and cytosolic access to individual neurons in the brain slice. The circuit elements (pipette resistance R_P, access resistance R_A, membrane resistance $R_{Membrane}$, and cell capacitance C_{cell}) are shown only for the whole-cell configuration. The intermediate cell-attached configuration (gigaseal formed but membrane patch unruptured) is shown on the bottom. Created with BioRender.

Supplementary Figure 2. SynGO enrichment across single neurons. (A) biological processes (BP) and (B) cellular component (CC) gene ontology (GO) terms across single-cell samples. A core set of 45 BP and 23 CC terms significantly enriched in all 12 neurons, indicating reproducible detection of synaptic proteins. GSEA Q-value significance cutoff < 0.05

Supplementary Figure 3. GPCR detection across single neurons. Binary heatmap showing presence (blue) or absence (white) of G protein-coupled receptors (GPCR) identified in individual neurons. Samples are annotated by the level of electrophysiological characterization (top bar). Green denotes whole-cell configuration was “Preserved”. Orange denotes whole-cell configuration was “Lost” during retrieval. Gray denotes whole-cell configuration was lost due to the neuron being “Torn” during retrieval. Red denotes no electrophysiological characterization due to a failure to form a gigaseal during the initial patch attempt. Clustering was performed by samples (columns) only.

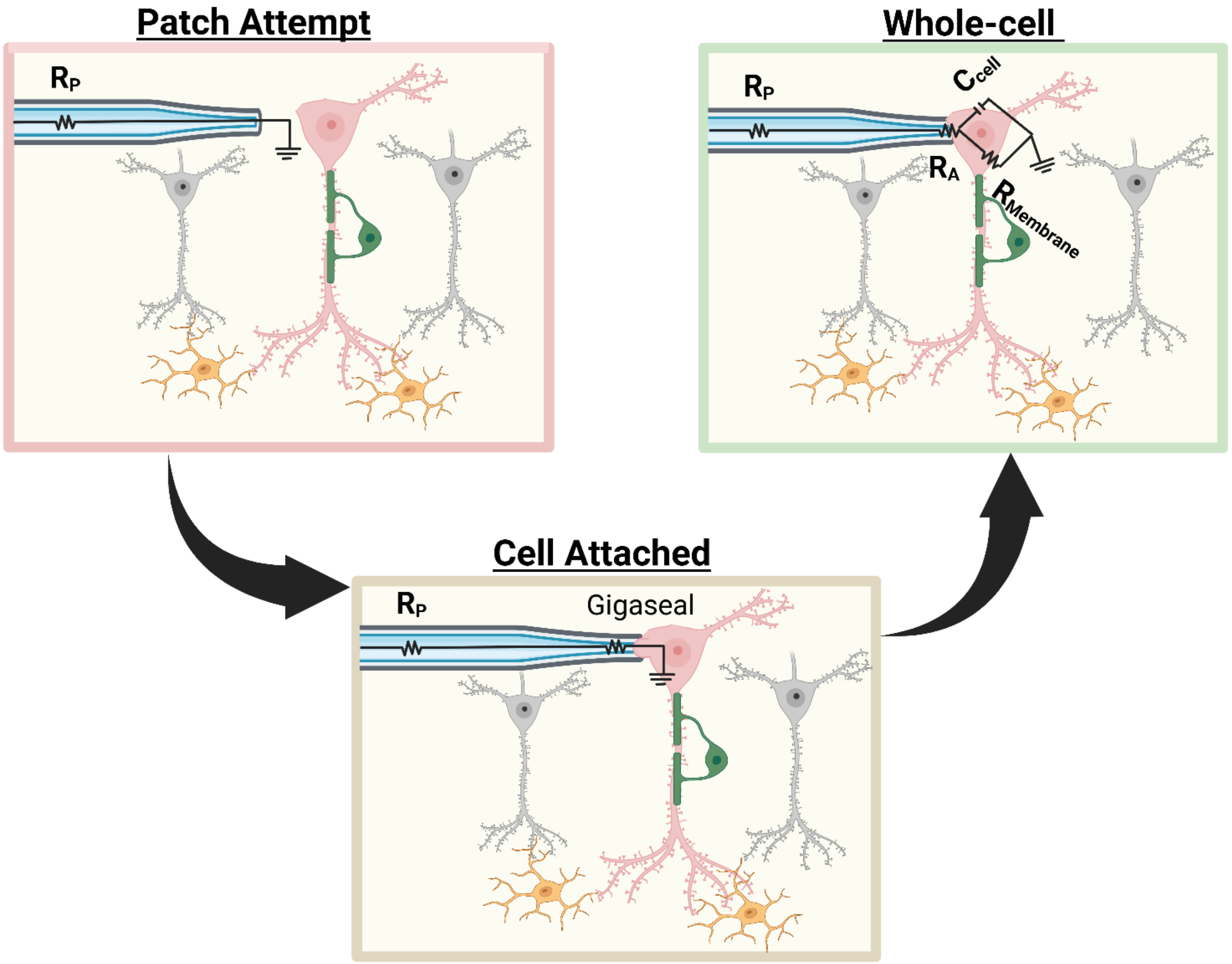

Supplementary Figure 1. Schematic of the patch-clamp process. Diagram illustrating the transition from initial pipette contact ("Patch attempt", top left) to full whole-cell configuration (top right), which allows simultaneous electrical and cytosolic access to individual neurons in the brain slice. The circuit elements (pipette resistance R_P, access resistance R_A, membrane resistance $R_{Membrane}$, and cell capacitance C_{cell}) are shown only for the whole-cell configuration. The intermediate cell-attached configuration (gigaseal formed but membrane patch unruptured) is shown on the bottom. Created with BioRender.

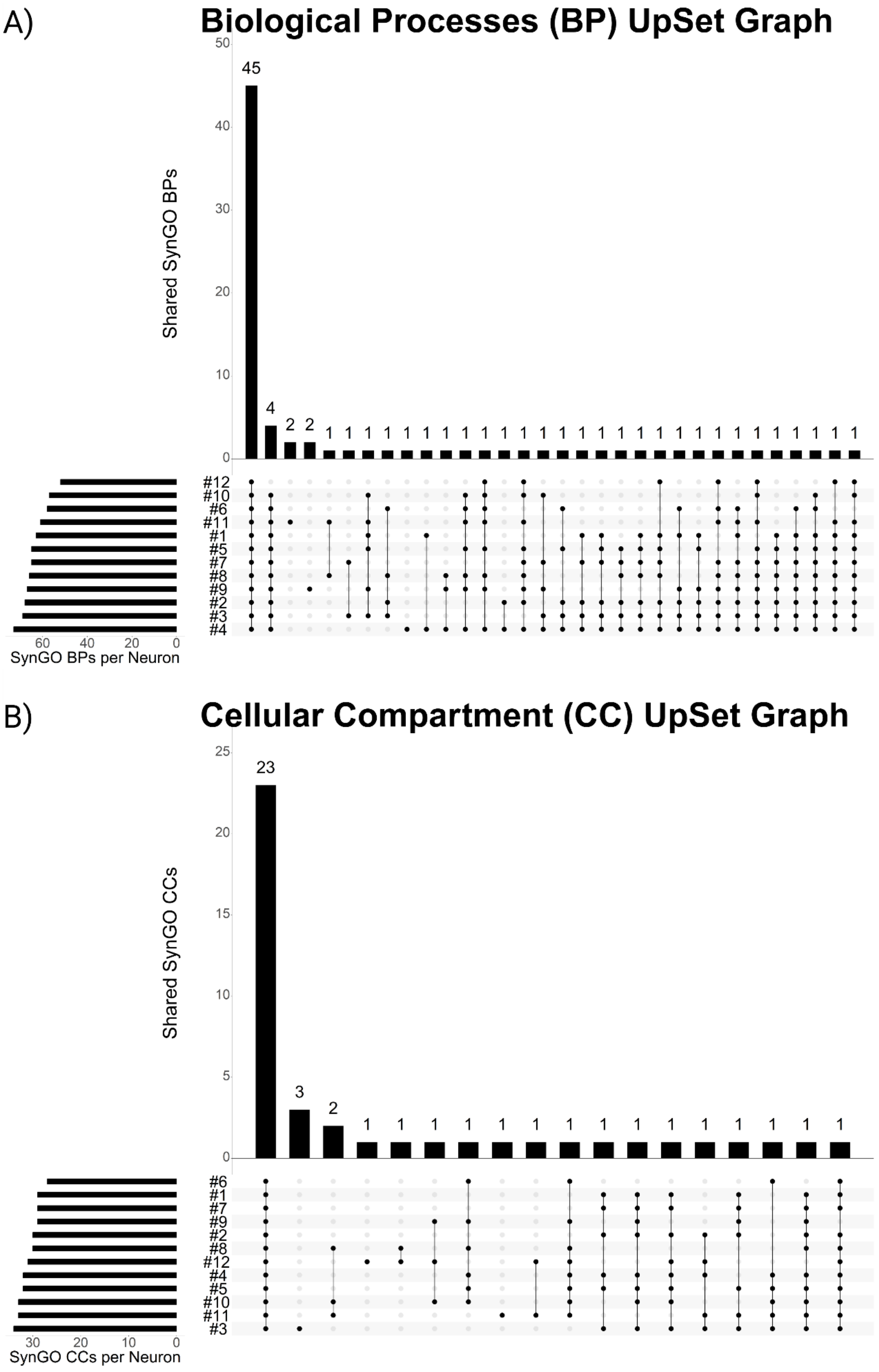
A)
Biological Processes (BP) UpSet Graph
Shared SynGO BPs
0
10
20
30
40
50
45
4
2
2
1 1 1 1 1 1 1 1 1 1 1 1 1 1 1 1 1 1 1 1 1 1 1 1 1 1 1 1
#12
#10
#6
#11
#1
#5
#7
#8
#9
#2
#3
#4
60
40
20
0
SynGO BPs per Neuron
B)
Cellular Compartment (CC) UpSet Graph
Shared SynGO CCs
0
5
10
15
20
25
23
3
2
1 1 1 1 1 1 1 1 1 1 1 1 1 1 1
#6
#1
#7
#9
#2
#8
#12
#4
#5
#10
#11
#3
30
20
10
0
SynGO CCs per Neuron

Supplementary Figure 2. SynGO enrichment across single neurons. (A) biological processes (BP) and (B) cellular component (CC) gene ontology (GO) terms across single-cell samples. A core set of 45 BP and 23 CC terms significantly enriched in all 12 neurons, indicating reproducible detection of synaptic proteins. GSEA Q-value significance cutoff < 0.05

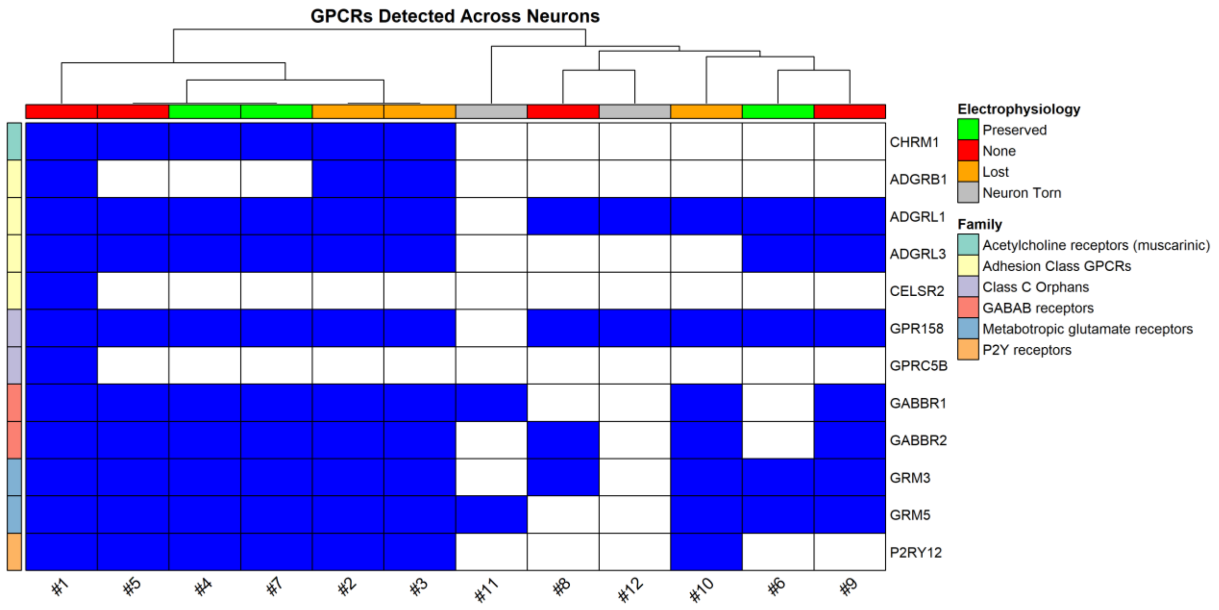

Supplementary Figure 3. GPCR detection across single neurons. Binary heatmap showing presence (blue) or absence (white) of G protein-coupled receptors (GPCR) identified in individual neurons. Samples are annotated by the level of electrophysiological characterization (top bar). Green denotes whole-cell configuration was "Preserved". Orange denotes whole-cell configuration was "Lost" during retrieval. Gray denotes whole-cell configuration was lost due to the neuron being "Torn" during retrieval. Red denotes no electrophysiological characterization due to a failure to form a gigaseal during the initial patch attempt. Clustering was performed by samples (columns) only.
