# Supplementary material for: Patch-Clamp Single-Cell Proteomics in Acute Brain Slices: A Framework for Recording, Retrieval, and Interpretation
Source: bioRxiv. 2025 Sep 17:2025.09.15.675920. Preprint. [Version 1] doi: 10.1101/2025.09.15.675920 (PMC12458218; doi:10.1101/2025.09.15.675920)
Supplement: Supplementary file 1 [file NIHPP2025.09.15.675920V1-supplement-1.pdf]

bioRxiv preprint doi: https://doi.org/10.1101/2025.09.15.675920; this version posted September 17, 2025. The copyright holder for this preprint (which was not certified by peer review) is the author/funder, who has granted bioRxiv a license to display the preprint in perpetuity. It is made available under aCC-BY 4.0 International license.

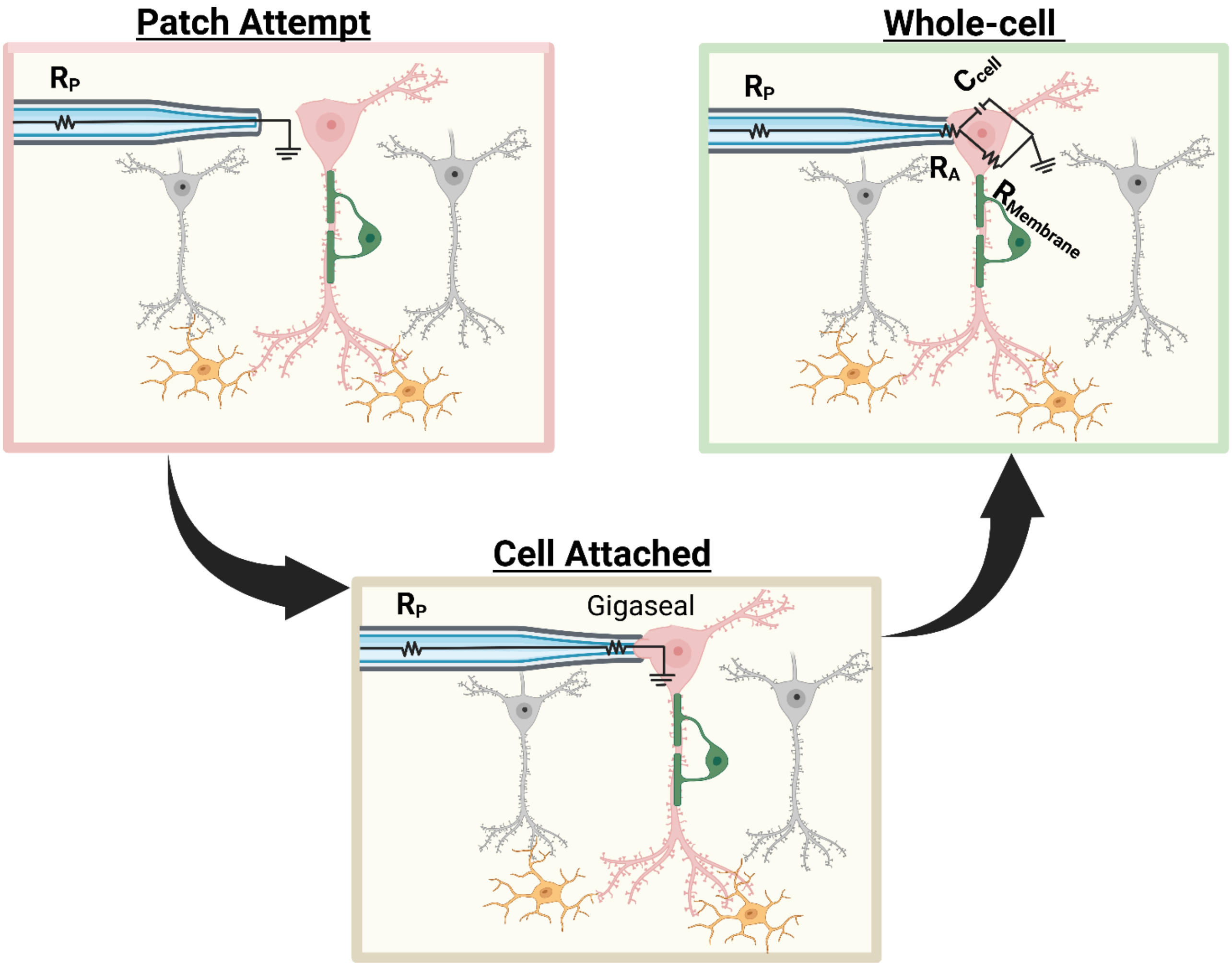

Supplementary Figure 1. Schematic of the patch-clamp process. Diagram illustrating the transition from initial pipette contact ("Patch attempt", top left) to full whole-cell configuration (top right), which allows simultaneous electrical and cytosolic access to individual neurons in the brain slice. The circuit elements (pipette resistance R_P, access resistance R_A, membrane resistance $R_{Membrane}$, and cell capacitance C_{cell}) are shown only for the whole-cell configuration. The

bioRxiv preprint doi: https://doi.org/10.1101/2025.09.15.675920; this version posted September 17, 2025. The copyright holder for this preprint (which was not certified by peer review) is the author/funder, who has granted bioRxiv a license to display the preprint in perpetuity. It is made available under aCC-BY 4.0 International license.

intermediate cell-attached configuration (gigaseal formed but membrane patch unruptured) is shown on the bottom. Created with BioRender.

bioRxiv preprint doi: https://doi.org/10.1101/2025.09.15.675920; this version posted September 17, 2025. The copyright holder for this preprint (which was not certified by peer review) is the author/funder, who has granted bioRxiv a license to display the preprint in perpetuity. It is made available under aCC-BY 4.0 International license.

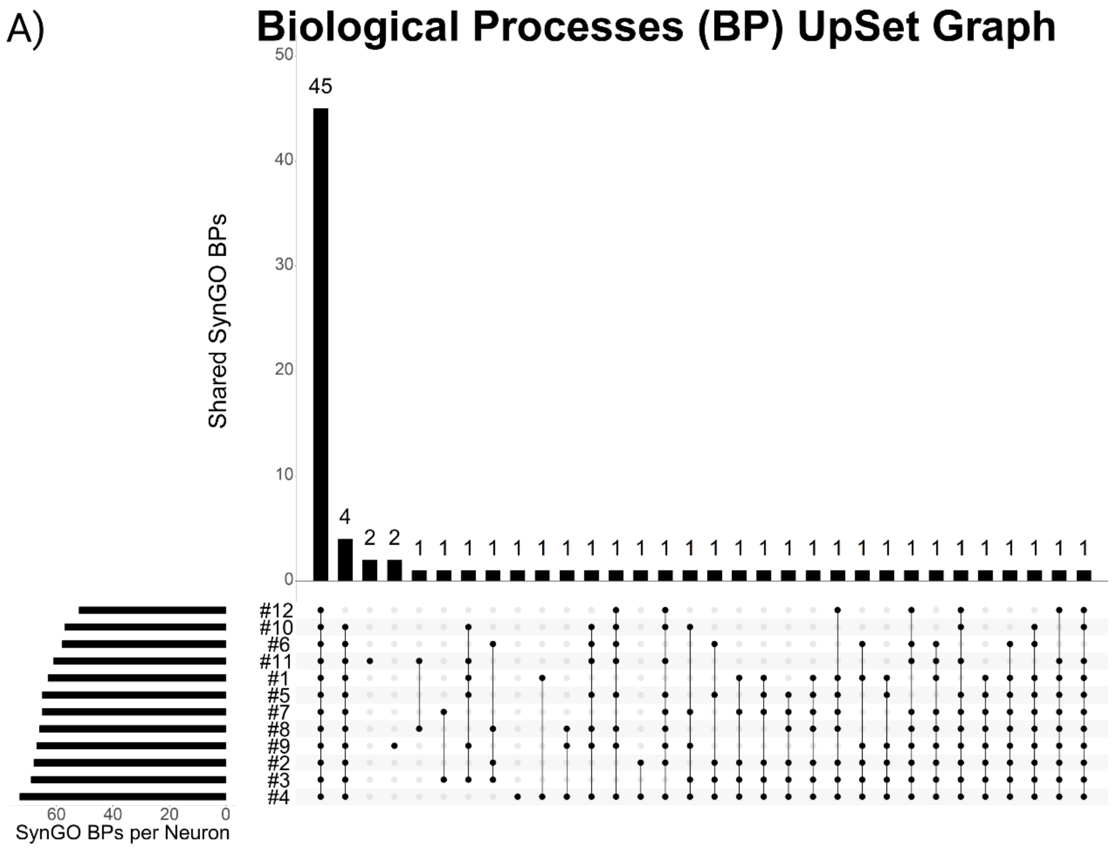

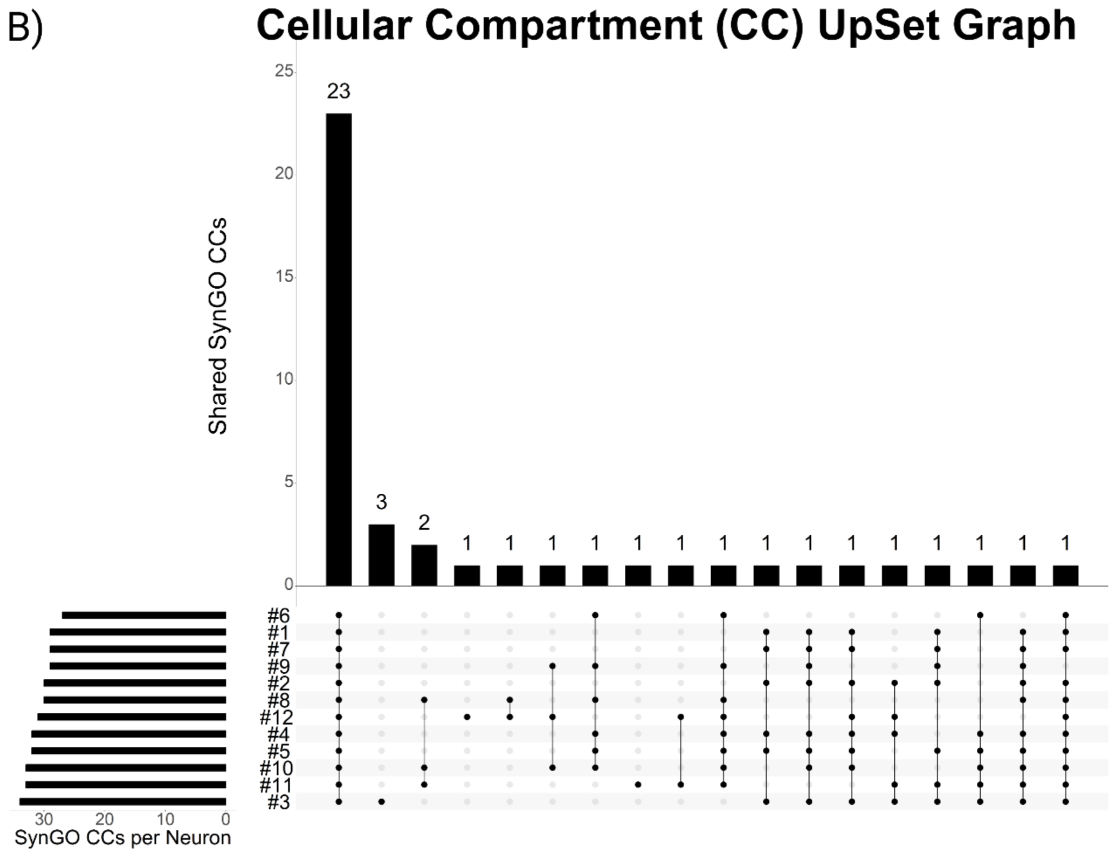

bioRxiv preprint doi: https://doi.org/10.1101/2025.09.15.675920; this version posted September 17, 2025. The copyright holder for this preprint (which was not certified by peer review) is the author/funder, who has granted bioRxiv a license to display the preprint in perpetuity. It is made available under aCC-BY 4.0 International license.

Supplementary Figure 2. SynGO enrichment across single neurons. (A) biological processes (BP) and (B) cellular component (CC) gene ontology (GO) terms across single-cell samples. A core set of 45 BP and 23 CC terms significantly enriched in all 12 neurons, indicating reproducible detection of synaptic proteins. GSEA Q-value significance cutoff < 0.05

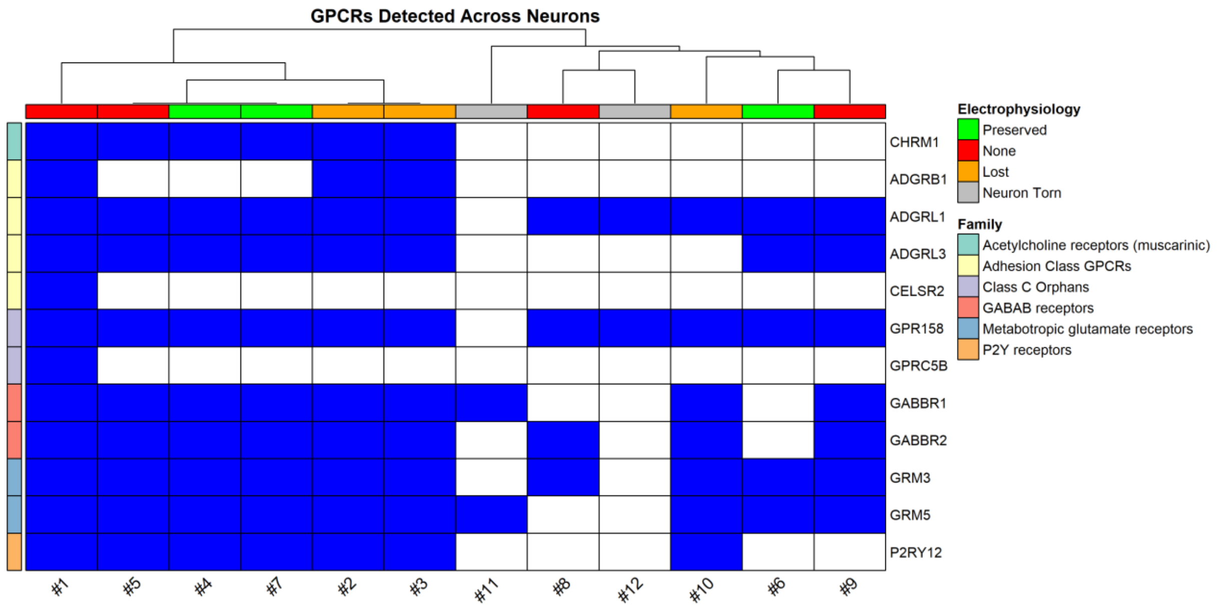

Supplementary Figure 3. GPCR detection across single neurons. Binary heatmap showing presence (blue) or absence (white) of G protein-coupled receptors (GPCR) identified in individual neurons. Samples are annotated by the level of electrophysiological characterization (top bar). Green denotes whole-cell configuration was "Preserved". Orange denotes whole-cell configuration was "Lost" during retrieval. Gray denotes whole-cell configuration was lost due to the neuron being "Torn" during retrieval. Red denotes no electrophysiological characterization due to a failure to form a gigaseal during the initial patch attempt. Clustering was performed by samples (columns) only.
